# Specific gut microbiota may increase the risk of erectile dysfunction: a two-sample Mendelian randomization study

**DOI:** 10.3389/fendo.2023.1216746

**Published:** 2023-12-18

**Authors:** Quanxin Su, Yanxi Long, Yayin Luo, Tao Jiang, Lei Zheng, Kenan Wang, Qizhen Tang

**Affiliations:** ^1^ Department of Urology, The First Affiliated Hospital of Dalian Medical University, Dalian, Liaoning, China; ^2^ Department of Neurology, The First Affiliated Hospital of Dalian Medical University, Dalian, Liaoning, China; ^3^ Department of Andrology, The Second Affiliated Hospital of Dalian Medical University, Dalian, Liaoning, China

**Keywords:** erectile dysfunction, gut microbiota, Mendelian randomization, prevention, diagnosis

## Abstract

**Objective:**

Studies have found that gut microbiota may be associated with the development of erectile dysfunction (ED); however, the exact link between the two remains unclear. This study aimed to elucidate the relationship between the gut microbiota and the risk of ED from a genetic perspective.

**Methods:**

We investigated the relationship between the gut microflora and ED using two-sample Mendelian randomization. GWAS-pooled data for ED were obtained from 223805 participants in Europe. GWAS summary data for ED were obtained from 223805 subjects in Europe and that for the gut microbiota were obtained from 18340 participants in 24 cohorts. We used the inverse-variance weighted (IVW) estimator as the primary method for the preliminary analysis, and the MR-Egger, weighted median (WM), simple model, and weighted model as secondary methods. We used Cochrane’s Q-test, to detect heterogeneity, MREgger to detect pleiotropy, and the leave-one-out method to test the stability of the MR results. Ultimately, we genetically predicted a causal relationship between 211 gut microbiota and ED.

**Results:**

A total of 2818 SNPs associated with gut microflora were screened in the ED correlation analysis based on the assumption of instrumental variables. The results of MR analysis showed a causal relationship between the six gut microbes and ED occurrence. The results of the fixed effects IVW method revealed five gut microflora, including Lachnospiraceae (OR, 1.265; P = 0.008), Lachnospiraceae NC2004 group (OR, 1.188; P = 0.019), Oscillibacter (OR, 1.200; P = 0.015), Senegalimassilia (OR, 1.355; P = 0.002), Tyzzerella3 (OR, 1.133; P = 0.022), to be negatively associated with ED. In addition, the IVW method revealed Ruminococcaceae UCG-013 (OR, 0.827; P = 0.049) to be positively associated with ED. Quality control results showed no heterogeneity or horizontal pleiotropy in the MR analysis (P > 0.05).

**Conclusions:**

Six gut microbes were genetically associated with ED; of which, Ruminococcaceae UCG-013 was causally associated with a reduced risk of ED development. Our findings provide a new direction for research on the prevention and treatment of ED; however, the mechanisms and details require further investigation.

## 1 Introduction

ED is a global health problem that seriously affects the physical and mental health of patients and their sexual partners, leading to depression, anxiety, and other psychiatric disorders, as well as affecting the sexual harmony of couples and family stability. Epidemiological surveys have shown that approximately 150 million males worldwide have varying degrees of ED, and the number of people with ED is estimated to increase to 300 million by 2025 (1–3). Inflammation plays an important role in ED development. Damaged endothelial cells stimulate an inflammatory response in the vessel wall by increasing the production of inflammatory factors and cell adhesion molecules, leading to the formation of atheromatous plaques in penile blood vessels (4, 5).

The intestinal flora is involved in regulating the metabolic, immune, endocrine, neurological, and other local and systemic physiological processes of the human body through its metabolites and derivatives, which greatly expand the metabolic capacity of the human body and is called the “second brain” (6). An impaired intestinal barrier and bacterial translocation caused by intestinal flora dysbiosis can promote the production of inflammatory cytokines and lead to systemic inflammation, which may further accelerate the progression of ED and cardiovascular diseases. Previous studies that targeted the intestinal flora to investigate the pathogenesis of ED were mainly based on observational cross-sectional analyses, and such results may be invalidated by confounding factors and cannot accurately reflect the causal relationship between intestinal flora and ED (7, 8).

Mendelian randomization was used to assess causality. The purpose was to estimate the causal relationship between exposure and outcome by modeling instrumental variables (IV) with genetic variants (such as single nucleotide polymorphisms [SNPs]) that are strongly associated with exposure (9). According to Mendel’s law of inheritance “parental alleles are randomly assigned to offspring,” the process of gamete formation in MR is therefore similar to a “natural” randomized controlled trial, largely excluding the interference of unobserved confounding factors. MR is widely used as an efficient and accurate method to investigate the causal relationship between health risk factors and disease outcomes (10–12).

In this study, we performed a two-sample MR analysis to investigate the causal relationship between the gut microflora and ED risk.

## 2 Methods

### 2.1 Study design

To explore the relationship between the gut microflora and ED at the genetic level, a two-sample Mendelian randomization approach was used in this study.

### 2.2 Data source

GWAS summary statistics for the gut microbiota were obtained from a large GWAS study by Kurilshikov et al. (MiBioGen Consortium, www.MiBioGen.org), which analyzed 18340 individuals from 24 cohorts and recorded 211 gut microbiota and 122110 associated SNPs (13). Summary statistics for ED were downloaded from the MRC IEU OpenGWAS dataset (GWAS ID:ebi-a-GCST006956) as outcome variables. This GWAS included data from 6175 patients and 217630 controls (14).

### 2.3 Selection of instrumental variables

As instrumental variables, three basic requirements need to be met: first, genetic variants must be strongly associated with exposure (in this case, gut microbiota); second, these genetic variants must be independent of any confounding factors in the exposure-outcome association; and third, genetic variants should not affect the outcome (in this case, ED) unless an association through exposure is possible.

To investigate the above hypothesis, the following conditions were met for SNP screening: first, SNPs were extracted from GWAS data related to gut microbiota (that is, exposure) using P < 1 × 10−5 as a screening criterion to demonstrate a strong association with exposure. Second, SNPs containing linkage disequilibrium (LD) were removed by running the ‘TwoSampleMR’ package with r2 = 0.001 and kb = 10000. Third, we searched the ED-related database for the SNPs corresponding to the gut microflora and the non-corresponding or palindromic SNPs were deleted. After the initial screening of SNPs, we calculated the F-value of each IV to exclude bias caused by weak IVs. IVs were considered weak when the F-value was greater than 10.

### 2.4 Estimation of causal effects

We used the inverse variance weighted method (IVW) to estimate the causal effect between the gut microbiota and ED. When heterogeneity was present, the random-effects IVW method was adopted. When no heterogeneity was observed, the fixed effects IVW method was used. In addition, we performed additional analyses using MR-Egger, weighted median method, simple model, and weighted model. Sufficient evidence of a causal effect was consistently provided by statistically significant IVW results and the direction of the results across all five analyses.

### 2.5 Quality controls

Heterogeneity refers to the presence of variability among the included studies. We used IVW model estimation to calculate heterogeneity and Q test for heterogeneity and P > 0.05 was used to indicate the absence of heterogeneity among the IVs. Horizontal pleiotropy refers to the phenomenon by which IVs influence outcomes through pathways other than exposure and is a potential source of bias. We examined the pleiotropy of the IVS using the MR-Egger regression method when P value was >0.05, which represented the absence of horizontal pleiotropy between the IVs and outcomes. Confounding factors may affect the results through multiple mechanisms dominated by the inclusion of genetic variants. Therefore, we used leave-one-out sensitivity analysis for IVs to determine whether the presence of a single SNP strongly influenced the results of the two-sample Mendelian randomization analysis.

The statistical analysis in this study was conducted using RStudio software (version 4.1.2). The resources used were mainly obtained from the TwoSampleMR R package developed by Hemani et al.

## 3 Results

### 3.1 Screening and validation of IVs

We screened the instrumental variables for 211 microflora and obtained 5,548,018 exposure-related instrumental variables. After removing chain imbalance effects, excluding variables weakly associated with exposure factors (F < 10), and using the online analysis software PhenoScanner to exclude variables that might be associated with outcome confounders (15), 2818 IVs from 211 microflora were finally included in the analysis.

### 3.2 Calculation of causal effects

Six causal associations between gut microbiota and ED risk were identified in this study. A higher genetically predicted family Lachnospiraceae, genus Lachnospiraceae NC2004 group, genus Oscillibacter, genus Senegalimassilia, genus Tyzzerella3 were associated with a higher risk of ED. In contrast, the genus Ruminococcaceae UCG-013 was associated with a lower risk.

#### 3.2.1 Lachnospiraceae

Through a series of quality controls, we obtained 17 SNPs as IVs for the MR analysis of Lachnospiraceae and ED, with one palindromic SNP (rs11755180) removed during quality control. Information on the instrumental variables is provided in **Supplementary Table 1**.

**Figure 1:**
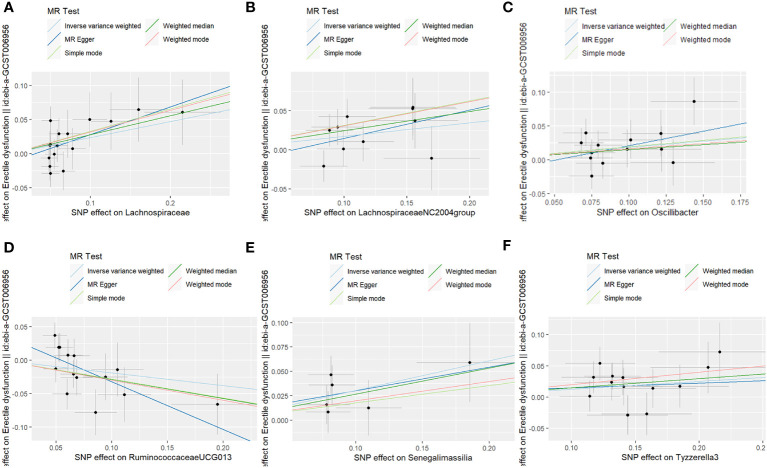
**(A–F)** The causality of different gut microbiota on ED risk.

The fixed-effects IVW results indicated that Lachnospiraceae (P = 0.008; OR, 1.265) negatively correlated with ED. The weighted median analysis showed similar results (P = 0.032; OR, 1.320). MR-Egger and simple model analyses indicated that Lachnospiraceae was not genetically causally related to ED (P > 0.05). The results of this analysis are presented in **Table 1** and **Figure 1**.

**Table 1:** Results of two-sample MR.

Exposure	Outcome	SNP	MR method	OR	95% LCI	95% UCI	p-value
Lachnospiraceae	ED	17	Inverse variance weighted (fixed effects)	1.265036	1.060802	1.508592	0.008869
			MR Egger	1.499954	0.954309	2.357581	0.099253
			Weighted median	1.32074	1.024017	1.703442	0.032129
			Simple mode	1.38725	0.889764	2.162893	0.167885
			Weighted mode	1.37399	0.990461	1.906031	0.075247
LachnospiraceaeNC2004group	ED	10	Inverse variance weighted (fixed effects)	1.188941	1.028684	1.374163	0.019137
			MR Egger	1.437936	0.775271	2.667015	0.282432
			Weighted median	1.2793	1.056047	1.549749	0.011825
			Simple mode	1.369055	1.009211	1.857204	0.07425
			Weighted mode	1.362182	0.978763	1.895803	0.100063
Oscillibacter	ED	13	Inverse variance weighted (fixed effects)	1.200767	1.035199	1.392815	0.015649
			MR Egger	1.536299	0.808229	2.920232	0.2168
			Weighted median	1.157483	0.942869	1.420948	0.162187
			Simple mode	1.209577	0.822125	1.77963	0.353202
			Weighted mode	1.17037	0.835145	1.640153	0.378867
RuminococcaceaeUCG013	ED	14	Inverse variance weighted (fixed effects)	0.827813	0.685696	0.999385	0.049252
			MR Egger	0.490894	0.276512	0.871489	0.031749
			Weighted median	0.751856	0.576771	0.980091	0.034972
			Simple mode	0.754557	0.463678	1.227913	0.277445
			Weighted mode	0.741449	0.509127	1.079783	0.142818
Senegalimassilia	ED	6	Inverse variance weighted (fixed effects)	1.355641	1.113311	1.650719	0.002459
			MR Egger	1.273835	0.605572	2.679544	0.558202
			Weighted median	1.306417	1.014469	1.682382	0.038328
			Simple mode	1.194321	0.817506	1.744823	0.400656
			Weighted mode	1.219242	0.846686	1.755729	0.335385
Tyzzerella3	ED	14	Inverse variance weighted (fixed effects)	1.133561	1.017835	1.262444	0.022504
			MR Egger	1.079292	0.585919	1.988109	0.810729
			Weighted median	1.159079	1.003664	1.338561	0.044454
			Simple mode	1.219764	0.938488	1.585341	0.161298
			Weighted mode	1.219764	0.953921	1.559692	0.137236

**Figure 2:**
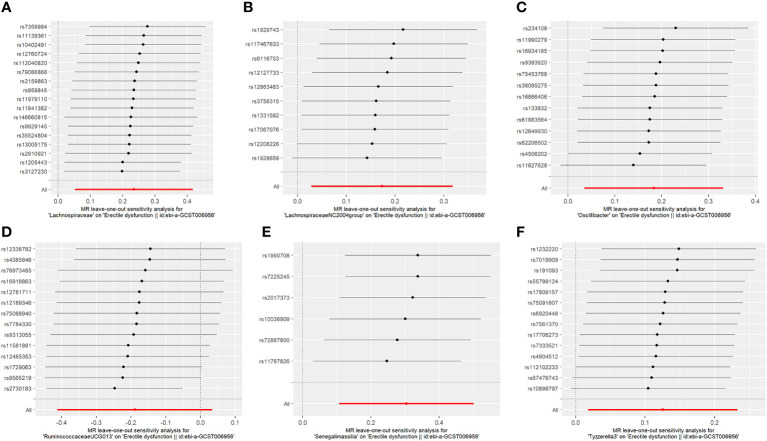
**(A–F)** The causality of each SNP of different gut microbiota on ED risk.

The results of the MR-IVW test showed no heterogeneity in the results of our MR analysis (P = 0.347, Q = 16.534). The results of the MR-Egger test for horizontal multiplicity indicated no horizontal multiplicity in our MR analysis (P = 0.431, SE = 0.015). The results of the sensitivity analysis are shown in **Figure 2**, which indicates that there was no particular SNP that would change the results, and that the final result was stable.

#### 3.2.2 LachnospiraceaeNC2004group

A total of 10 SNPs were included in the MR analysis of the Lachnospiraceae NC2004 group and ED, with no palindromic SNPs removed (**Supplementary Table 2**). The fixed-effects IVW results indicated that Lachnospiraceae (P = 0.019; OR, 1.188) negatively correlated with ED. The results for w were similar to those for IVW (P = 0.011; OR, 1.279). MR-Egger and simple model analyses indicated that Lachnospiraceae was not genetically causally related to ED (P > 0.05). MR-IVW and Q tests revealed the absence of horizontal pleiotropy and heterogeneity. No single SNP outliers were identified using the leave-one-out method.

#### 3.2.3 Oscillibacter

Fourteen SNPs were included in the analysis after quality control, including three palindromic SNPs (rs12417956, rs137917150, and rs6901560) (**Supplement Table 3**). The fixed-effects IVW results indicated that Oscillibacter (P = 0.015; OR, 1.200) had a negative causal relationship with ED. The other four analysis methods indicated that Oscillibacter was not causally related to ED (P > 0.05). No significant heterogeneity or horizontal pleiotropy was found according to the Cochrane’s Q and MR-Egger tests (**Supplement Table 7**). When the SNPs were removed individually, the results remained stable (**Figure 2C**).

#### 3.2.4 Ruminococcaceae UCG-013

Fourteen SNPs were included in the analysis after quality control, with one palindromic SNP (rs2428106) removed (**Supplement Table 4**). Fixed effects IVW, MR-Egger, and weighted median results indicated that Ruminococcaceae UCG-013 had a positive causal relationship with ED. The simple and weighted models did not consider the relationship between Ruminococcaceae UCG-013 and ED. Quality control analysis revealed no significant heterogeneity or horizontal pleiotropy.

#### 3.2.5 Senegalimassilia

Six SNPs were included in the analysis after quality control, including two palindromic SNPs (rs13383270 and rs57512504) (**Supplementary Table 5**). The results of the fixed effects IVW, MR-Egger, and weighted median analyses indicated that Senegalimassilia had a negative causal relationship with ED (P = 0.002 vs. P = 0.038). The MR-Egger, simple, and weighted models showed no association with ED. No significant heterogeneity or horizontal pleiotropy was found in quality control.

#### 3.2.6 Tyzzerella3

After quality control, 14 SNPs were included in the analysis. No palindromic SNPs were identified (**Supplementary Table 6**). Using the IVW method, Tyzzerella3 was found to have a negative genetic association with ED (P = 0.022; OR=1.133). The other four analytical methods indicated that Tyzzerella3 had no causal genetic relationship with ED. In addition, the results of the MR-Egger tests confirmed that there was no horizontal pleiotropy (P = 0.875, SE = 0.045), and the results of the Cochrane Q-test showed that there was no notable heterogeneity among the selected SNPs (P = 0.584, Q = 11.312). In addition, no outlier SNPs were identified after applying the leave-one-out method.

## 4 Discussion

Common risk factors for ED include age, diabetes, dyslipidemia, hypertension, cardiovascular disease, obesity, metabolic syndrome, hyperhomocysteinaemia, physical inactivity, and smoking (16–18). The intestinal microflora is closely related to the development, growth, and metabolism of the host, and a stable intestinal flora environment plays an important role in regulating the immune response of the body (19). The composition and metabolites of intestinal flora can be influenced by external environmental factors. If the normal intestinal environment is disturbed, the gut is unable to function as an antimicrobial agent and the release of inflammatory factors in the gut increases intestinal permeability, leading to increased levels of inflammation and oxidative stress, and this proinflammatory state leads to endothelial cell dysfunction (20). It is common in the pathogenesis of cardiovascular diseases and type 2 diabetes, and the development of ED is closely linked to the pathology of these diseases. Therefore, we hypothesized that an association exists between the intestinal flora and its metabolites and the development of ED.

To our knowledge, this is the first large-scale, comprehensive MR study to examine the causative role of gut microbes in ED. The advantage of two-sample Mendelian randomization studies over traditional single-sample studies is that they can be analyzed using aggregated GWAS data, thus extending the range of available data (21–23). According to the core principles of Mendelian randomization, studies conducted in this manner can largely avoid the effects of reverse causality and can effectively avoid bias from confounding factors that cannot be controlled for in traditional observational studies of the ED (including smoking, alcohol consumption, and obesity associated with the ED). The quality control results showed no heterogeneity or horizontal multiplicity for any outcome. Based on the principle of MR method selection, that is, preferentially using IVW fixed-effects model estimates in the absence of heterogeneity and multiplicity, and preferentially using results calculated using the MR-Egger method in the presence of multiplicity, we finally adopted the IVW fixed-effects model estimates as the primary study outcomes (24). We identified six microbial communities associated with ED occurrence; of which, five (Lachnospiraceae, Lachnospiraceae NC2004 group, Oscillibacter, Senegalimassilia, and Tyzzerella3) were associated with an increased risk of ED, and one (Ruminococcaceae UCG-013) was associated with a decreased risk of ED.

At the family level, Lachnospiraceae, which colonizes the intestinal lumen from birth and increases in number, is part of the core gut microbiota. Several studies have shown that a high abundance of Lachnospiraceae positively correlates with glucose and/or lipid metabolism, indicating metabolic disorders (25, 26). Metabolic syndromes, including arterial hypertension, insulin resistance, and hypertriglyceridemia, significantly increase the risk of developing diabetes and cardiovascular diseases (27) and are the risk factors for ED. In a study by Zhang et al., a significant increase in Lachnospiraceae was observed in the feces of hyperlipidemic mice (28). One study reported that different OTUs of Lachnospiraceae were associated with changes in lipid metabolism and specific nutrients such as saturated and total fats, and thus obesity (29). In addition, a positive correlation exists between the different taxa of Lachnospiraceae (particularly Anaerostipes, Bhatia, Dorea, and Lachnospiraceae incertae sedis) and major depression (30–32), whereas depression and other emotional or psychiatric disorders have been shown to trigger ED (33, 34). Therefore, we speculate that Lachnospiraceae may increase the risk of ED by affecting blood lipid levels and interfering with neurotransmission in the central nervous system (35).

In addition, at the genus level, Oscillibacter, Tyzzerella 3, and Senegalimus ssilia were associated with an increased risk of ED development. Barandouzi et al. found an increased abundance of Oscillibacter in the feces of patients with depression (36). Kelly et al. found that an increased abundance of Tyzzerella 4 may be a risk factor for cardiovascular disease progression (37). Wang et al. found that Senegalimassilia spp. was associated with increased blood pressure (38). These findings suggest that gut microbes have important effects on vascular endothelial function, depression, and cognitive function in humans, thereby increasing the risk of ED development. Interestingly, gut flora may play different roles in different ethnic groups. Liu et al. analyzed the relationship between the gut microbiome and blood metabolites in an Asian population and found that Oscillibacter was causally linked to decreased triglyceride concentrations (39), suggesting that Oscillibacter may play a role in reducing ED risk in Asian populations; however, these results need to be validated in future studies.

Our genetic prediction-based study also found a strong causal relationship between Ruminococcaceae and reduced risk of ED (OR, 1.13; 95% CI, 1.02–1.25; P = 0.02). Combined with the results of previous studies, the targeted modulation of bacterial abundance appears to be a novel approach for reducing the risk of ED. Feng et al. found that Ruminococcaceae_UCG-013 was more abundant in the gut microbes of lean mice than in obese mice. Further analysis revealed that Ruminococcaceae UCG-013 was positively correlated with serum HDL-C levels and negatively correlated with serum TC, TG, and LDL-C levels. This suggests that an increased abundance of Ruminococcaceae lowers total blood cholesterol levels, thereby reducing the risk of ED (40).

Hyperhomocysteinemia is an emerging risk factor for ED. Folic acid and vitamin B12 supplements are commonly used in clinical practice to reduce HCY levels (41, 42). Studies have shown that some gut microbiota, such as red bacteria and bifidobacteria, can synthesize B vitamins and folic acid, which, to some extent, complements the human body’s requirements for folic acid and B vitamins. We speculate that gut microbiota may also influence the incidence of ED.

In addition, the current treatment of ED mainly includes oral drugs, psychotherapy and other methods. Although there are many treatment methods for ED, there are also problems such as the treatment rate is difficult to further improve. At present, probiotics and fecal bacteria transplantation and other treatment methods related to intestinal flora have been used to regulate glucose and lipid metabolism and improve vascular endothelial injury. Therefore, the treatment of ED by regulating the intestinal flora is a potential therapeutic method in the future, which is worthy of further exploration. Our results provide a potential target for the treatment of ED by regulating the intestinal flora. In the follow-up, we will further explore the specific mechanism of these six flora in ED.

This study has some limitations. First, the data in this study were all from European populations, and whether the conclusions can be generalized to other populations requires further validation. Second, this study was only a statistical result and could not further explore the biological mechanism between the gut microbiota and ED. Third, this study lacked data from subgroups stratified by age, previous surgical information and hormone levels and could not compare the causal effect differences between subgroups.

## 5 Conclusion

This study assessed the causal relationship between the gut microbiota and ED in 211 people using two-sample Mendelian randomization. The results showed that Ruminococcaceae UCG-013 may reduce the risk of ED. Lachnospiraceae and Tyzzerella3 may increase the risk of ED. Our study provides a new research direction for preventing and treating ED. We plan to further investigate the specific relationship between these gut microbes and ED and explore their potential value in the pathogenesis of ED.

## Data availability statement

The original contributions presented in the study are included in the article/**Supplementary Material**. Further inquiries can be directed to the corresponding authors.

## Ethics statement

This study was partly based on the publicly available data. It did not include interaction with humans or use personal identifying information. Thus, the informed consent for this study was not required.

## Author contributions

Conceptualization: QT. Data curation: QT, QS. Formal analysis: QS. Funding acquisition: QT, QS. Methodology: QT, LZ. Supervision: QT, TJ. Validation: KW, YYL. Writing-original draft: QS. Writing – review & editing: TJ, YXL. All authors contributed to the article and approved the submitted version.
