# Supplementary material for: Specific gut microbiota may increase the risk of erectile dysfunction: a two-sample Mendelian randomization study
Source: Front Endocrinol (Lausanne). 2023 Dec 18;14:1216746. doi: 10.3389/fendo.2023.1216746 (PMC10773840; doi:10.3389/fendo.2023.1216746)
Supplement: Supplementary file 1 [file Table_1.docx]

**Supplementary Table1**: SNP information of Lachnospiraceae.

| SNP | Chr | Pos | EA | NEA | Beta | SE | P | F |
| --- | --- | --- | --- | --- | --- | --- | --- | --- |
| rs9929145 | 16 | 76559184 | G | A | -0.12572 | 0.024523 | 2.84E-07 | 26.28195 |
| rs959845 | 4 | 186906801 | C | T | -0.04937 | 0.01078 | 5.17E-06 | 20.97095 |
| rs79086868 | 9 | 133791605 | T | C | 0.077672 | 0.016447 | 3.01E-06 | 22.30218 |
| rs7359994 | 19 | 29408858 | T | C | -0.05031 | 0.011248 | 5.36E-06 | 20.00865 |
| rs35524804 | 9 | 100113683 | T | C | -0.06075 | 0.012522 | 2.45E-06 | 23.5368 |
| rs3127230 | 10 | 103392503 | C | T | -0.05035 | 0.01122 | 6.20E-06 | 20.14058 |
| rs2910921 | 5 | 32348593 | T | C | 0.160302 | 0.035832 | 8.42E-06 | 20.01409 |
| rs2159863 | 4 | 10245354 | A | G | -0.05858 | 0.012874 | 3.70E-06 | 20.70635 |
| rs146660815 | 6 | 46664437 | T | C | -0.21511 | 0.047678 | 6.53E-06 | 20.35658 |
| rs13005175 | 2 | 232469722 | A | G | 0.099348 | 0.02184 | 8.37E-06 | 20.69357 |
| rs12760724 | 1 | 214031969 | A | C | -0.04846 | 0.010793 | 7.27E-06 | 20.15529 |
| rs1205443 | 20 | 36876868 | A | G | 0.050142 | 0.011216 | 7.29E-06 | 19.98666 |
| rs11979110 | 7 | 130436459 | T | C | -0.05009 | 0.010507 | 1.82E-06 | 22.72988 |
| rs11841382 | 13 | 38004672 | G | T | -0.07181 | 0.017462 | 9.58E-06 | 16.91287 |
| rs112040820 | 17 | 80469064 | A | G | 0.054958 | 0.011709 | 2.42E-06 | 22.03234 |
| rs11139361 | 9 | 72179847 | T | C | 0.049446 | 0.01105 | 4.26E-06 | 20.02243 |
| rs10402491 | 19 | 13425799 | C | T | 0.06617 | 0.014851 | 7.58E-06 | 19.85248 |

**Supplementary Table2**: SNP information of LachnospiraceaeNC2004group.

| SNP | Chr | Pos | EA | NEA | Beta | SE | P | F |
| --- | --- | --- | --- | --- | --- | --- | --- | --- |
| rs6116753 | 20 | 5330700 | G | A | 0.099475 | 0.020914 | 2.92E-06 | 22.62326 |
| rs3756315 | 5 | 149544722 | A | G | -0.08835 | 0.01884 | 3.33E-06 | 21.99055 |
| rs1929743 | 13 | 76829562 | T | C | 0.083721 | 0.019032 | 9.06E-06 | 19.35098 |
| rs1928659 | 9 | 29334137 | T | C | 0.102522 | 0.022645 | 6.17E-06 | 20.49781 |
| rs17067076 | 18 | 58229086 | G | A | -0.15463 | 0.035219 | 5.61E-06 | 19.27667 |
| rs1331592 | 9 | 122239986 | C | G | 0.094881 | 0.020837 | 5.34E-06 | 20.73432 |
| rs12863463 | 13 | 47512191 | G | A | -0.15636 | 0.034537 | 6.04E-06 | 20.49772 |
| rs12208226 | 6 | 22725908 | C | A | -0.15474 | 0.034037 | 9.75E-06 | 20.66769 |
| rs12127733 | 1 | 234157721 | G | A | 0.115182 | 0.024601 | 3.11E-06 | 21.92167 |
| rs117467633 | 17 | 21157406 | T | C | -0.16969 | 0.038317 | 9.13E-06 | 19.61191 |

**Supplementary Table3**: SNP information of Oscillibacter.

| SNP | Chr | Pos | EA | NEA | Beta | SE | P | F |
| --- | --- | --- | --- | --- | --- | --- | --- | --- |
| rs9393920 | 6 | 28580593 | A | G | -0.07447 | 0.015108 | 9.92E-07 | 24.29394 |
| rs761240 | 20 | 49507892 | T | G | -0.17664 | 0.038881 | 2.04E-06 | 20.63938 |
| rs75453768 | 10 | 116469806 | G | T | 0.12212 | 0.026863 | 5.35E-06 | 20.66649 |
| rs62206502 | 20 | 18003390 | C | A | -0.06814 | 0.015135 | 6.60E-06 | 20.271 |
| rs61883564 | 11 | 7901384 | A | G | -0.10135 | 0.022102 | 3.39E-06 | 21.02876 |
| rs4506202 | 8 | 21598077? | A | G | -0.07113 | 0.015226 | 3.21E-06 | 21.8251 |
| rs36095275 | 14 | 32270129 | C | T | -0.07524 | 0.015686 | 1.40E-06 | 23.00544 |
| rs234108 | 1 | 184942671 | A | G | 0.074955 | 0.015263 | 9.16E-07 | 24.11644 |
| rs16934185 | 9 | 1798324 | A | G | -0.12957 | 0.028157 | 4.38E-06 | 21.17481 |
| rs16866406 | 2 | 179457147 | A | G | 0.098877 | 0.02088 | 3.08E-06 | 22.42562 |
| rs133832 | 22 | 44834707 | A | C | -0.07955 | 0.016241 | 1.15E-06 | 23.9928 |
| rs12649930 | 4 | 3656291 | T | G | 0.121589 | 0.025961 | 4.09E-06 | 21.93501 |
| rs11990279 | 8 | 11116314 | T | C | -0.08249 | 0.018046 | 4.94E-06 | 20.89723 |
| rs11627628 | 14 | 21479605 | T | C | 0.143961 | 0.029022 | 1.01E-06 | 24.60504 |

**Supplementary Table4**: SNP information of RuminococcaceaeUCG013.

| SNP | Chr | Pos | EA | NEA | Beta | SE | P | F |
| --- | --- | --- | --- | --- | --- | --- | --- | --- |
| rs9565219 | 13 | 76583319 | T | A | -0.05246 | 0.01177 | 8.73E-06 | 19.8646 |
| rs9313055 | 5 | 3628882 | T | C | 0.105087 | 0.023446 | 9.55E-06 | 20.08916 |
| rs7784330 | 7 | 32530997 | G | A | -0.04983 | 0.011207 | 8.16E-06 | 19.77355 |
| rs76973485 | 3 | 9534657 | G | T | 0.194976 | 0.041821 | 3.35E-06 | 21.73527 |
| rs75088940 | 12 | 30245557 | T | C | -0.0943 | 0.020071 | 2.55E-06 | 22.07218 |
| rs4385846 | 10 | 115650278 | G | T | 0.05984 | 0.013181 | 6.46E-06 | 20.61168 |
| rs2730183 | 8 | 40311885 | G | A | -0.04887 | 0.010991 | 8.44E-06 | 19.77041 |
| rs1729063 | 16 | 10863459 | G | C | -0.05334 | 0.012076 | 9.64E-06 | 19.51206 |
| rs16918863 | 10 | 19780463 | A | C | 0.111491 | 0.024016 | 4.16E-06 | 21.55216 |
| rs12781711 | 10 | 2219930 | C | T | -0.06561 | 0.011748 | 2.55E-08 | 31.19435 |
| rs12485353 | 3 | 197049996 | G | A | -0.06079 | 0.013085 | 4.19E-06 | 21.58443 |
| rs12336782 | 9 | 13609119 | T | C | -0.0856 | 0.018931 | 8.60E-06 | 20.44768 |
| rs12189346 | 5 | 141395140 | G | A | 0.068496 | 0.014558 | 1.68E-06 | 22.13738 |
| rs11581881 | 1 | 9361576 | C | T | 0.066121 | 0.014474 | 4.73E-06 | 20.87006 |

**Supplementary Table5**: SNP information of Senegalimassilia.

| SNP | Chr | Pos | EA | NEA | Beta | SE | P | F |
| --- | --- | --- | --- | --- | --- | --- | --- | --- |
| rs72887800 | 17 | 74949284 | T | A | -0.08225 | 0.017572 | 2.42E-06 | 21.90713 |
| rs7225245 | 17 | 48379615 | G | A | 0.079173 | 0.017042 | 4.18E-06 | 21.58282 |
| rs2017373 | 14 | 0.6262 | C | T | 0.078226 | 0.017684 | 9.50E-06 | 19.56748 |
| rs1990708 | 2 | 2.07E+08 | A | C | -0.10962 | 0.024779 | 8.91E-06 | 19.57153 |
| rs11787826 | 9 | 34332382 | C | A | 0.081327 | 0.017115 | 2.63E-06 | 22.57849 |
| rs10036909 | 5 | 1.28E+08 | C | T | 0.185519 | 0.040088 | 8.05E-06 | 21.41647 |

**Supplementary Table6**: SNP information of Tyzzerella3.

| SNP | Chr | Pos | EA | NEA | Beta | SE | P | F |
| --- | --- | --- | --- | --- | --- | --- | --- | --- |
| rs7561370 | 2 | 57810406 | T | C | 0.131341 | 0.028629 | 1.52E-06 | 21.04659 |
| rs75091807 | 13 | 35023691 | G | T | -0.18497 | 0.038303 | 1.71E-06 | 23.31961 |
| rs7333521 | 13 | 81593016 | T | C | -0.20719 | 0.045312 | 4.88E-06 | 20.90818 |
| rs7019909 | 9 | 33113322 | T | C | 0.144156 | 0.030163 | 1.76E-06 | 22.84145 |
| rs6920448 | 6 | 5755626 | C | T | -0.14108 | 0.030545 | 4.15E-06 | 21.3337 |
| rs67476743 | 19 | 1030320 | T | G | 0.132164 | 0.022208 | 3.74E-09 | 35.41653 |
| rs55799124 | 17 | 3738781 | A | G | -0.11435 | 0.02386 | 1.34E-06 | 22.96767 |
| rs4904512 | 14 | 89595945 | T | C | -0.11715 | 0.025031 | 3.09E-06 | 21.90535 |
| rs191093 | 12 | 77390329 | G | A | 0.159008 | 0.035331 | 6.76E-06 | 20.25523 |
| rs17809157 | 7 | 82547827 | A | T | -0.16382 | 0.033639 | 1.54E-06 | 23.7167 |
| rs17706273 | 5 | 16388259 | T | C | -0.14037 | 0.027471 | 5.88E-07 | 26.10937 |
| rs1232220 | 6 | 102680257 | G | T | -0.14387 | 0.031828 | 7.91E-06 | 20.43226 |
| rs112102233 | 10 | 46098286 | A | G | -0.21635 | 0.047758 | 6.18E-06 | 20.5222 |
| rs10898797 | 11 | 87588698 | C | T | 0.12238 | 0.027468 | 8.85E-06 | 19.85002 |

**Supplementary Table7**: Results of Cochran's Q test.

| Exposure | Outcome | method | Q | Q_df | Q_pval |
| --- | --- | --- | --- | --- | --- |
| Lachnospiraceae | ED | MR Egger | 16.53473 | 15 | 0.34743 |
|  |  | IVW | 17.25612 | 16 | 0.369214 |
| LachnospiraceaeNC2004group | ED | MR Egger | 7.092396 | 8 | 0.526698 |
|  |  | IVW | 7.477515 | 9 | 0.587531 |
| Oscillibacter | ED | MR Egger | 10.47571 | 11 | 0.488172 |
|  |  | IVW | 11.07303 | 12 | 0.522671 |
| RuminococcaceaeUCG013 | ED | MR Egger | 13.9525 | 12 | 0.303752 |
|  |  | IVW | 18.18467 | 13 | 0.150633 |
| Senegalimassilia | ED | MR Egger | 2.777474 | 4 | 0.595727 |
|  |  | IVW | 2.806419 | 5 | 0.7298 |
| Tyzzerella3 | ED | MR Egger | 11.28709 | 12 | 0.504489 |
|  |  | IVW | 11.31266 | 13 | 0.584643 |

**Supplementary Table8**: Results of pleiotropy.

| Exposure | Outcome | Egger_intercept | SE | pval |
| --- | --- | --- | --- | --- |
| Lachnospiraceae | ED | -0.01245895 | 0.015401 | 0.431177 |
| LachnospiraceaeNC2004group | ED | -0.02167074 | 0.03492 | 0.552141 |
| Oscillibacter | ED | -0.02237031 | 0.028945 | 0.455888 |
| RuminococcaceaeUCG013 | ED | 0.03914674 | 0.020519 | 0.08062 |
| Senegalimassilia | ED | 0.005947428 | 0.034958 | 0.873165 |
| Tyzzerella3 | ED | 0.007197548 | 0.04501 | 0.875614 |
